# Texting to Increase Physical Activity Among Teenagers (TXT Me!): Rationale, Design, and Methods Proposal

**DOI:** 10.2196/resprot.3074

**Published:** 2014-03-12

**Authors:** Debbe Thompson, Dora Cantu, Riddhi Bhatt, Tom Baranowski, Wendy Rodgers, Russell Jago, Barbara Anderson, Yan Liu, Jason A Mendoza, Ramsey Tapia, Richard Buday

**Affiliations:** ^1^USDA/ARS Children's Nutrition Research CenterPediatricsBaylor College of MedicineHouston, TXUnited States; ^2^Department of Family and Community MedicineBaylor College of MedicineHouston, TXUnited States; ^3^Physical Education and RecreationUniversity of AlbertaEdmonton, ABCanada; ^4^Centre for Exercise, Nutrition & Health SciencesSchool for Policy StudiesUniversity of BristolBristolUnited Kingdom; ^5^PsychologyDepartment of PediatricsBaylor College of MedicineHouston, TXUnited States; ^6^Seattle Children's Research InstituteUniversity of WashingtonSeattle, WAUnited States; ^7^Archimage, LLCHouston, TXUnited States

**Keywords:** physical activity, pedometers, text messages, self-determination theory, adolescents

## Abstract

**Background:**

Physical activity decreases from childhood through adulthood. Among youth, teenagers (teens) achieve the lowest levels of physical activity, and high school age youth are particularly at risk of inactivity. Effective methods are needed to increase youth physical activity in a way that can be maintained through adulthood. Because teens text a great deal, text messages promoting walking, a low cost physical activity, may be an effective method for promoting sustainable physical activity.

**Objective:**

The objective of our study was to determine the effect of pedometers, self selected step goals, and texts grounded in the self-determination theory (SDT) on physical activity among the teens.

**Methods:**

“TXT Me!” was a 12 week intervention that texted 14-17 year olds to increase their daily physical activity by increasing the number of steps they take each day. The intervention was grounded in the SDT. Formative research with the teens helped construct the intervention and develop the texts. A total of 84 texts were developed (12 to set a step goal, and 72 promoting autonomy, competence, and relatedness). The pilot evaluation used a four group, randomized design (n=160). After baseline data collection, the participants were randomized to one of four conditions (no treatment control, pedometer only, pedometer + weekly prompts, pedometer + weekly prompts + SDT grounded texts). Data were collected at baseline and immediately upon completion of the study. The primary outcome was physical activity, measured by 7 days of accelerometry. Basic psychological needs, physical activity motivation, process evaluation, and program satisfaction data were also collected.

**Results:**

To our knowledge, this is one of the first studies to explore the use of stand alone, SDT grounded texts, supported by pedometers and prompts to set a self selected step goal, as a method for increasing physical activity among teens.

**Conclusions:**

This pilot study will contribute valuable information regarding whether theoretically grounded text messages show promise as an effective method to increase physical activity among teens.

**Trial Registration:**

Clinicaltrials.gov NCT01482234; http://clinicaltrials.gov/ct2/show/NCT01482234 (Archived by WebCite at http://www.webcitation.org/6NYvRMOoq).

## Introduction

### Obesity and Chronic Diseases

Obesity has reached epidemic proportions both in the United States and other countries [1,2]. This is a major public health concern primarily because obesity increases the risk of developing chronic diseases [3], such as certain cancers [4,5], cardiovascular disease [6], and type 2 diabetes mellitus [7]. Obese adolescents are more likely to become obese adults [8], thus increasing current and future risk of chronic disease. Therefore, preventing obesity during adolescence would be of major public health significance.

### Decreased Physical Activity in Adolescence

Physical activity (PA) has been inversely related to adiposity [9] and decreases risk for cardiovascular disease [10], type 2 diabetes [11], and certain cancers [12]. However, research demonstrates that PA steadily decreases from childhood through adolescence [13]. Between the ages of 6 and 16, PA decreased approximately 50%, with a continued decrease into adulthood [14]. Between early and late adolescence, moderate to vigorous PA decreased substantially among both boys (6.7 hours per week to 5.1 hours per week) and girls (5.9 hours per week to 3.5 hours per week) [15], suggesting that adolescence is a particularly vulnerable time for PA. Finding ways to avoid this decline would have a significant impact on adolescent PA and obesity risk.

Walking is a popular form of PA [16]. Walking is convenient, inexpensive, and can be easily incorporated into everyday life [17,18] in multiple ways (eg, leisure time PA, active transport) [19]. Increased walking is likely a sustainable form of PA that could be maintained over time [20]. Interventions that encourage simple activities such as walking could make substantial inroads into increasing sustainable PA [18]. Brisk walking is equivalent to moderate intensity PA [21-24]. Given the potential public health significance of walking [18], research is needed to identify effective methods for promoting walking in groups at risk of low levels of PA, such as adolescents.

Pedometers monitor daily steps, thus providing a convenient way to set goals, self monitor PA, and serve as a reminder to be active [25]. Pedometer use has been associated with increased PA in adults [26] and youth [27]. Having a step goal has been associated with decreased body mass index in adults [26]. Among youth, pedometers alone may not be enough to increase PA; other strategies to augment pedometers may be needed to promote sustained PA [27]. As there is a shortage of youth pedometer based studies, there is a need to identify effective methods for incorporating pedometers into interventions promoting walking and to evaluate the long term effectiveness of these interventions [27].

### Texting and Teenagers

Cellphone ownership and texting are high among teenagers (teens) [28]. A recent study found that 77% of 12-17 year olds had a cell phone, and that texting is the primary way 14-17 year olds communicate with others (eg, friends, family) [28]. Teens are prolific users of SMS text messaging (short message service, SMS), with a median of 100 texts sent and received each day [28]. Since texting is a familiar, convenient, and acceptable way that teens communicate with others, texting may be an effective way to increase PA in this at risk group. A number of reviews of cell phone based studies suggest this intervention mode is promising [29-32]. Some characteristics of text message based interventions that appeared to enhance effectiveness were matching text message frequency to expected frequency of behavior (eg, daily PA), tailoring to selected characteristics (eg, personal values), and interactivity (eg, ability to communicate with research team) [31]. A limitation that was cited was that few of the randomized control trials using texts were theory based [32].

### The Self-Determination Theory

Interventions that are based on psychological theories increase the likelihood of achieving behavior change [33]. The self-determination theory (SDT) [34] posits that three basic psychological needs underpin behavior: (1) competence (ie, skills, ability), (2) autonomy (ie, choice, control), and (3) relatedness (ie, connection to self and others). Need satisfaction (as related to the SDT) integrates the behavior into one’s sense of self (ie, how one defines him/herself). The higher the need satisfaction, the greater the integration with sense of self, thus increasing motivation to perform the behavior, behavioral performance, and the likelihood the behavior will be maintained over time [34]. The SDT has guided PA focused studies in both youth [35-44] and adults [45]. A systematic review of adult studies concluded the evidence supported the SDT as a framework for identifying key factors that influence and promote exercise [45]. Promising evidence is also emerging from youth studies. The SDT predicted autonomous motivation and step counts in youth [36], as well as intention to engage in leisure time PA [37]. The SDT explained changes in motivation to engage in school physical education in the transition from elementary to middle school [40]. An intervention with teens that focused on need satisfaction (ie, autonomy, competence, relatedness) enhanced cardiovascular fitness and was well received by youth [35]. Satisfaction of the basic psychological needs was also associated with autonomous motivation and PA in children [42]. Therefore, the SDT informed interventions promoting PA to youth that emphasize the basic psychological needs might be of particular utility in enhancing motivation to engage in PA, and, ultimately, PA.

Behavioral interventions should be systematically developed and tested [46] to maximize resource effectiveness and increase the likelihood of success. Ideally, interventions should be focused on behaviors that can be changed, evidence based, appropriate for the target population, and focused on realistic, achievable goals [46]. This paper describes the rationale, design, development, and methods for a systematically developed pilot intervention using pedometers, prompts to set a step goal, and SDT grounded text messages promoting walking (ie, increased daily steps) to teens.

## Methods

### Overview

This research included two phases: (1) formative, to develop the intervention and construct the text messages, and (2) pilot to assess the feasibility of this approach. Both of these phases are described below.

### Participants

Teens for both phases of the research were recruited using the volunteer database at the Children’s Nutrition Research Center. The standard recruitment methods, (eg, newsletter and website announcements, distribution of flyers in community locations likely to be visited by parents and teens) were also used. The parents provided written informed consent for their teen to participate in the study, and teens provided written assent. The teens that participated in the formative phase of the program were not eligible to participate in the pilot study.

Eligible participants were 14-17 years old, fluent in English, with Internet access, an email address, and access to a cell phone with unlimited text messaging. Exclusionary criteria included mental (eg, learning impairments) or physical (eg, blindness, deafness, inability to be physically active, medical conditions that limited PA) conditions that impaired their ability to fully participate in the program and/or complete data collection. The families that were interested in participating in the study contacted the Children’s Nutrition Research Center’s Recruitment Coordinator. She explained the study in detail, screened interested families, and routed eligible teens to the study coordinator.

### Intervention

#### Focus

This program encouraged walking (eg, attaining a certain number of daily steps), a low cost PA that does not require special equipment, membership fees (eg, gym), or a high level of fitness [17,18]. It is also an activity that has the potential to be maintained over time [20].

#### Formative Research

There were thirty 14-17 year olds, stratified by gender (male, female) and race/ethnicity (black, Hispanic, white) that were invited to participate in two rounds of formative research. During each round of formative research, the teens completed a Web-based survey and then participated in a telephone interview with trained research coordinators to discuss the survey responses. Probes were used to clarify and understand responses and to ensure responses were interpreted as intended.

Formative research revealed that teens had positive reactions to wearing a pedometer to help them keep track of the number of steps they took each day and to receiving daily texts to help them attain a step goal. The teens thought that attaining 12,000-15,000 steps a day would be “easy.” Most of them wanted the study to set a step goal for them, and they wanted to text their daily steps to the study. The most common reasons teens wanted to be physically active were related to appearance (eg, lose weight, be fit, appeal to opposite gender) and sports/athletics. Most of them reported having unlimited texting plans and having to adhere to family (eg, no profanity, violence, sexting) and school (eg, no texting during class) rules about texting. They also reported talking with parents, friends, especially close friends, and others, such as coaches and teachers about PA. All the teens believed that teens have control over whether they are physically active, although the amount of control they exert over whether they are physically active varied from some to total control. Most thought 6 a.m.-8 a.m. would be the ideal time to receive texts about PA.

The teens suggested keeping the text messages short (<160 characters); ensuring messages were positive, straightforward, and promoted realistic behaviors; and conveyed emotion through the use of emoticons [47], (eg, :D) and exclamation points (!). Other suggestions included ensuring the texts did not nag (eg, “Don’t forget...you’re in charge of meeting your step goal.”), or sound like school (eg, “Having a problem meeting your step goal? Make a list of ways to get extra steps. You’ve got what it takes!”). The teens also suggested not using words like “problem solving” or “brain storming,” text abbreviations (eg, “gr8” for “great”), or suggestions that were unrealistic, unclear, or too formal. In other words, the teens wanted straightforward, realistic text messages that focused on facts.

There were two types of text messages that were developed: (1) reminders to set a weekly step goal (eg, prompts), and (2) SDT grounded text messages emphasizing the basic psychological needs (ie, autonomy, competence, relatedness). The texts were vetted by the teens and a professional panel of experts in psychology, behavior change, PA, and SDT prior to use in the pilot study. Examples of modifications after the texts were reviewed by the teens are presented in Textbox 1.

Autonomy was operationalized as having choice or control over PA, while competence focused on having the skills or abilities to meet step goals and be physically active. Relatedness was operationalized as a sense of connectedness to self and important others (ie, family, friends). Connectedness to self was promoted by relating step goal attainment to personal values [48,49]. During formative assessment, the teens were shown a list of personal values that was used in a previous intervention with youth [49]. The values receiving the highest ratings were; “being healthy and fit”, “being responsible”, “being successful”, “getting good grades”, and “being a good person”. The teens did not relate to messages attempting to connect meeting a daily step goal with “being a good person”. Further, since “healthy” and “fit” are slightly different concepts, they were split and promoted separately. The final values used to promote a sense of connectedness to self were; “being healthy”, “being fit”, “being responsible”, “being successful”, and “getting good grades”. Examples of how “relatedness to self” was operationalized are presented in Table 1.

Using the formative research described above as a guide, texts were <160 characters, straightforward, realistic, and conveyed emotion through emoticons and exclamation points, actual words, rather than text abbreviations, were used. A total of 84 texts were finalized for the pilot feasibility study, 12 prompts to set a step goal, and 72 SDT grounded texts. The SDT grounded texts were evenly divided between those promoting autonomy, competence, and relatedness. Relatedness was further divided into text messages promoting a connection with self (ie, personal values) and others (ie, friends, family). Prompts were sent once a week (Sunday), and SDT grounded texts were sent 6 times a week (Monday-Saturday).

**Sample messages after formative research with teens.**

*Modifications to a text prompt reminding teens to set a step goal-*

Original-“It’s that time again. What will your daily goal be this week?”

Teen reactions-Suggested adding an emoticon, shortening, and removing “it’s that time again.”

Revision-“What will your step goal be this week? :D”

 

*Modifications to a SDT grounded text promoting autonomy-*

Original-“You are in control of your life's choices; meeting your step goal today is your choice.”

Teen reactions-Liked this text, felt empowered; liked the implied conclusion; suggested shortening it.

Revision-“Your steps, your choice, your life! You're in charge!”

 

*Modifications to a SDT grounded text promoting competence-*

Original-“You are capable of using strategies like problem solving and self monitoring, to help you meet your step goal.”

Teen reactions-Wordy; sounds like school; unconversational.

Revision-“You are capable of using strategies to reach your step goal! Success is yours!”

**Table 1:** Texts connecting personal values to meeting step goal.

Personal value	Text message
Being healthy	“Meeting your step goal shows being healthy is important to you.”
Being fit	“’Step’ towards fitness. Meet your step goal today!!”
Being responsible	“Meeting your step goal shows you take responsibility for your health.”
Being successful	“Experience success! Meet your step goal today. :D”
Getting good grades	“Meeting your step goal shows you know how to achieve your goals, like getting good grades.”

#### Conceptual Model

**Figure 1:**
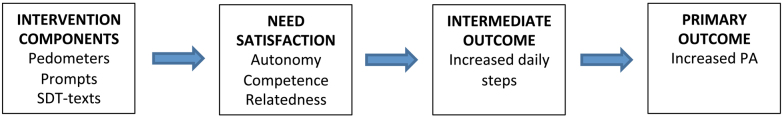
Conceptual model guiding the intervention. Self-determination theory (SDT); physical activity (PA).

Figure 1 shows a conceptual model of the intervention. The intervention components include pedometers (to self monitor, promotes competence), prompts (to set a self selected step goal, promotes autonomy), and SDT informed text messages emphasizing the basic psychological needs within the context of daily steps (promotes autonomy, competence, relatedness). Emphasizing the basic psychological needs in the context of taking more daily steps enhances motivation to take more daily steps, which leads to increased daily steps, and ultimately, increased PA. The pilot study tests the feasibility of this approach.

### Pilot Study Design

A four group design was employed: (1) no treatment control, (2) pedometer only, (3) pedometer + weekly texts to set a step goal (ie, prompts), and (4) pedometer + weekly texts to set a step goal + SDT focused text messages. This design is expected to provide insight into the additive effects of intervention components (pedometers, prompts, SDT focused text messages). Data were collected at baseline and upon completion of the 12 week intervention (post 1 data collection). The primary outcome was objectively measured PA. Randomization occurred after baseline data collection using a random numbers table. After obtaining written informed consent/assent, the participants began baseline data collection. Institutional review board approval was obtained from Baylor College of Medicine (H-27537). The study was registered with Clinicaltrials.gov prior to initiation (NCT01482234).

### Procedures

#### Intervention

Although the formative assessment with the teens indicated their desire to have the study set a daily step goal for them, the teens were not assigned a daily step goal by the study. Goals set by others are inconsistent with the SDT and the development of higher levels of PA motivation (ie, autonomous motivation) [34]; autonomous motivation is more likely to lead to sustained PA (ie, increased daily steps) [45]. Rather, each teen’s average daily step count was extracted from baseline activity monitor data. When they were notified of their group assignment and sent a pedometer, all the teens except those randomized to the control group were told that experts recommend teens attain a daily step count of 12,000 to 15,000 [50]. They were then given their average daily step count and told that increases should be gradual. This was deemed to be general knowledge instead of a goal, since it did not adhere to the characteristics of a goal (ie, specific, moderately difficult, temporal) [51], much like the recommendation to attain 60 or more minutes of PA a day [52]. The teens in the control group received their average daily baseline step count, a pedometer, and the recommended guideline at the end of the study.

A browser based administrative software application was developed to manage participants, send texts, and view messages received from participants. The management application enabled the research team to enter participants into their assigned group after randomization. A database of the text messages (ie, prompts and theory based messages) was created and an algorithm was developed to connect message type with group. Each morning at 8 a.m., the algorithm driven application was automatically executed to query the database by group, and identify whether texts were to be sent to each group that day, and if so, which texts to send. The application then communicated with an SMS gateway provider (Clickatel), via their Web-based application programming interface to automatically send the messages. Prompts were sent each Sunday, and SDT focused texts were sent Monday-Saturday.

An additional feature in the administrative application enabled preformatted messages to be manually texted to participants by the research team through the SMS gateway. Examples of preformatted messages included texts to let the teens know that it was time for data collection and/or that the activity monitor had been sent to them by prepaid courier service. These messages were texted to all participants on an as needed basis by the research staff, regardless of group assignment.

The teens could also send texts to the research team through the SMS gateway. Research staff could manually respond to texts received by the teens during the data collection or intervention period through the SMS gateway. Although the teens were not asked to text their step goal or step attainment to the research team because of its inconsistency with the SDT, some chose to text this information to the team anyway. Examples of other types of texts received from the teens included questions, texts to let the research team know they had received a package, or other types of general communication with the research team.

The administrative management application and the SMS gateway were hosted on secure servers and accessed via a Secure Socket Layer connection. An automatic log was maintained to track all activity of the application, which included both manual and automated messages sent by the research team, message delivery (both automated and manual), messages received from the study participants, and any technical issues encountered during message delivery (automated, manual).

#### Data Collection

Objectively measured PA was assessed with 7 days of accelerometry (Actigraph, Limited Liability Corporation; Model GT3X+). Self reported data included the Basic Psychological Need Satisfaction Questionnaire [53], PA motivation (intrinsic, extrinsic) as measured by the expanded Behavioral Regulation in Exercise Questionnaire [54], social desirability of response [55,56], program satisfaction (post 1 only), and standard demographics (baseline only), these data were collected using a secure, password protected website. Process data were collected using the framework of Baranowski and Jago [57]. The areas emphasized included recruitment, retention, and program delivery; process data were recorded in an Access database maintained by study staff. A log of technical issues was also maintained, as was a record of texts sent to and received from participants.

#### Beta Testing

Extensive internal beta testing was conducted with the research staff and programmers prior to the feasibility study. A one month test of the procedures and text messages with participants who participated in the formative research was also conducted and served as a final beta test of intervention, messages, and procedures prior to the feasibility study. No technical or procedural issues were identified during the one month beta test.

## Results

### Pilot Study

A 12 week pilot study was initiated in August 2012 and completed in August 2013. There were one hundred and sixty 14-17 year olds that were enrolled (40/group) using the volunteer database (ie, families interested in participating in research studies) at the Children’s Nutrition Research Center and also using standard recruitment procedures (posting of flyers, posting of study details on recruitment websites and/or electronic newsletters). The teens were randomized after completing a baseline data collection. The baseline characteristics indicated that 51.9% (83/160) were female, and 60.6% (97/160) were at the lower end of the age range (ie, less than 16 years old).  The sample was ethnically diverse (35.6%, 57/160 black; 31.3%, 50/160 white; 26.9%, 43/160 Hispanic; and 6.3%, 10/160 mixed/other), respectively.  The teens wore an activity monitor for 7 days to obtain an objective measure of PA; they also completed Web-based self report questionnaires. Baseline psychometric values of the measurement scales (Cronbach alpha) were within acceptable ranges [58] (Psychological Need Satisfaction in Exercise, .87; expanded Behavioral Regulation in Exercise Questionnaire, .84). Data are currently being analyzed to determine feasibility of this approach to promote PA to teens.

### Expected Outcomes

Although the pilot study was not powered to detect statistically significant differences, the group receiving pedometers + weekly prompts + SDT text messages is expected to have the greatest increase in moderate to vigorous PA and average daily steps. They are also expected to have the greatest increases in basic psychological needs, and the greatest movement towards higher levels of PA motivation. The group receiving pedometers + weekly prompts is expected to have the next greatest increase in moderate to vigorous PA, steps, psychological needs, and PA motivation, followed by the group receiving pedometers only. The control group is expected to exhibit no change in moderate to vigorous PA, steps, basic psychological needs, or PA motivation. It is also expected that recruitment goals will be met, attrition will be low, and that we will be able to collect complete data (Web-based self report, 7 days of accelerometry) in at least 75.0% (120/160) of study participants at post 1, regardless of group assignment. It is also anticipated that few technical issues will limit distribution of SMS text messaging; that program satisfaction will be high among participants, particularly in the group that received daily messages; and that the internal consistency of self report measures will be acceptable (>.70).

## Discussion

### Teenagers and Physical Activity

Teens are at risk of low levels of PA [15]. This is a major public health concern because low levels of PA, particularly during adolescence, have been shown to track into adulthood [14], thus increasing the risk of obesity [9] and multiple chronic diseases [10-12]. Effective interventions are needed to increase PA among this at risk group. Because walking is an activity that can be relatively easily incorporated into one’s other daily activities [17,18], interventions encouraging teens to take more steps throughout the day may be an effective way to enhance sustainable PA in this at risk group.

### An Intervention for Teenagers

The intervention described here provides teens with a pedometer to self monitor their daily steps, encourages them to set a self endorsed daily step goal through once a week texts, and sends daily texts emphasizing the basic psychological needs. It extends the literature in several important ways. First, it systematically varies the intervention components (none, pedometers only, pedometers + prompts, pedometers + prompts + SDT informed texts), which will provide information on whether adding prompts or SDT informed text messages increase daily steps and PA over pedometers alone. Second, it will add to the body of literature on whether SDT informed interventions are an effective way to promote PA among teens. Text messages promoting obesity prevention behaviors have been shown to be acceptable and feasible with obese teens; however, their effect on behavior was not assessed, and they were developed as an adjunct to a weight management program rather than as a stand alone intervention [59]. The current research extends this by examining intervention effects on daily steps; specifically, it examines whether an SDT informed text message based intervention focusing on the basic psychological needs is feasible, acceptable, and influences average daily steps, motivation, and PA in teens. Third, it adds further support to the importance of conducting formative research with teens to help ensure the intervention meets their expectations [60]. Ultimately, this could enhance intervention effectiveness by designing interventions that are developmentally appropriate and relevant to the target audience, an important lesson learned for researchers conducting research with teens. Fourth, it extends the evidence regarding the relationship between pedometers and PA in teens [27]. And finally, it extends the literature on texting as a stand alone intervention modality versus as a supporting component. Although reviews provide suggestive evidence that text messages may be an effective way to promote health enhancing behaviors [29-32], few have examined it as a stand alone intervention method. A text message based intervention to increase PA in teens showed promising results; however, PA was self reported, and the intervention only lasted two weeks [61]. The current study will extend this research by using a stronger measure of PA (objectively measured), and sending text messages for 12 weeks.

### Conclusions

Teens are heavy users of texting [62]. Therefore, a text based intervention encouraging teens to take more steps during the day may be an effective way to encourage them to be more physically active in a manner that has the potential to be sustained throughout life [20]. This is an important area of research; a text message based intervention would be easy to disseminate at a relatively low cost, using a familiar and convenient technology. Thus, this research ultimately has the potential for public health significance by increasing PA in an at risk group in a familiar, convenient, and relatively low cost manner.

## Abbreviations

PA: physical activity
SDT: self-determination theory
SMS: short message service
teens: teenagers
USDA/ARS: United States Department of Agriculture, Agricultural Research Service
